# Treatment of Bronchopleural Fistula with Carbolic Acid instilled through Bronchofiberscope in post-pulmonectomy patients

**DOI:** 10.1186/s13019-015-0316-8

**Published:** 2015-09-15

**Authors:** Zheng Wang, Han-Bing Yu, Quan Luo, Yong-Yu Liu

**Affiliations:** Department of Thoracic Surgery, Liaoning Tumor Hospital, Xiaoheyan Road No.44, Dadong District, Shenyang, 110042 P.R. China

**Keywords:** Carbolic acid, Bronchopleural fistula, Bronchofiberoscope

## Abstract

**Objective:**

To investigate the efficacy of carbolic acid treatment of bronchopleural fistula (BPF) using bronchofiberscope (BFS) in post-pulmonectomy patients.

**Method:**

Twelve patients with post-pulmonectomy BPF were enrolled in this study at the Liaoning Tumor Hospital between February 2009 and March 2012. Three patients had BPF after the right pneumonectomy, six patients after left pneumonectomy, one patient after the right middle and low lobectomy and two patients after left upper lobectomy. BPF patients were instilled with 100 % carbolic acid (0.5–1 ml one time every week) through BFS on the mucosal surface around the fistula, and the bubble disappearance was monitored. Treatment was repeated if the bubble remained.

**Results:**

No haemorrhage, severe dyspnea or SpO2 declines occurred in all the 12 patients during the bronchoscopic therapy. BPF orifices were closed in five patients after receiving 5 treatments with carbolic acid, 1 patient received 2 treatments, 1 patient was given 3 treatments, 2 patients received 4 treatments and 3 patients were given 7 treatments. Follow-up was conducted for six months following bronchoscopy. The average treatment and fistula closure time were calculated from the data collected as 20 min and 30 days, respectively, and the cure rate was 100 %. Hematoxylin-eosin (HE) staining results revealed that the white flat hyperplasia tissue after carbolic acid treatment was inflammatory granulation tissue.

**Conclusion:**

Our results revealed that instillation of 100 % carbolic acid with BFS to treat BPF was 100 % effective, which can be a support for post-pulmonectomy BPF.

## Background

Bronchopleural fistula (BPF) is a relatively infrequent but potentially fatal complication of pulmonary resection. BPF can be divided into peripheral or central, based on the location of the leakage, and BPF occurs in about 1.5 to 28 % of pneumonectomy cases, and is associated with high death rate [9, 30]. It is estimated that incidence of BPF after pneumonectomy and lobectomy for lung cancer is 4.5–20 % and 0.5 %, respectively, and the incidence of BPF is highest after right pulmonary resection and right lower lobectomy [31]. The etiology of BPF includes incomplete tumor resection, use of steroids, intraoperative infection and prolonged postoperative mechanical ventilation as major risk factors of BPF [31]. The clinical manifestations of BPF can be frequently classified as acute, subacute, and chronic. An acute BPF presents as tension pneumothorax, with pleural cavity communicating abnormally with the airways, and is associated with purulent sputum expectoration, dyspnea, and reduction in established pleural effusion [22]. The presentations of subacute and chronic BPF are commonly related to a pleural space with infection, manifesting as a more invisible form with fever, dry cough, and malaise with different levels of respiratory disorder [33]. Traditional treatments of BPF include thoracotomy after drainage and primary repair, which is based on vascularized muscular flaps and omental grafts tissues [20]. Amplatzer vascular plug, which was originally designed for the transcatheter closure of vascular structures, has also been reported as a safe and effective method to treat small postoperative BPF [9]. Fruchter et al. also found that the technique of Amplatzer double-disk occluder implantation may be suitable for both large and small BPFs which originate from the main bronchi and lobar bronchi, respectively [8]. Additionally, endoscopic approaches and bronchoscopy are common methods of treating BPF to avoid thoracotomy [27, 36].

Bronchofiberscope (BFS) is a precision instrument employed to diagnose bronchial diseases using of the light guide composed by the fine fibers formed by tens of thousands of high transmittance glass or acrylic resin [12, 16]. BFS is designed to offer advantageous features such as easy operation method, clear vision, mild trauma, tolerance of surgery by patients, and high safety profile, which reduces or avoids complications associated with tracheotomy and prevents local infection [25, 32]. Clinically, BFS has multiple uses, including removing foreign bodies, eliminating secretions, treating nasopharyngeal carcinoma, central lung cancer, alveolar cell carcinoma, esophageal fistula, hemoptysis, obstruction, assisting endotracheal intubation treatment and placing gastric tube [1, 11, 21]. Previous studies have revealed that BFS is also an excellent diagnostic tool for early detection of various intrabronchial injuries, and the attached biopsy sampling feature is helpful in the identification of early lesions, and to carry out poly excision surgery for the studies on bronchus and lung diseases [15, 18, 19]. Previous studies reported various treatment methods for BPF using BFS, and the methods include gelfoam, shot put plugs, and tissue adhesives. However, these methods have significant deficiencies, evident from the fact that treatment fistula under 3 mm was efficient using these methods, but they show poor efficacy in treatment of BPF beyond 3 mm, particularly those beyond 10 mm [6, 28, 35, 37]. Phenol, also named carbolic acid, is a sweet-smelling colorless liquid used to prepare resins, preservatives, fungicides, drugs (e.g., aspirin), and also is used to disinfect surgical instruments [4, 24, 38]. 88 % carbolic acid was found to be efficacious with all alopecia areata patients and can be considered as a treatment of choice for stable alopecia areata [3]. Moreover, spot peel with 88 % phenol can be a cost-effective procedure for idiopathic guttate hypomelanosis, which can be combined with other medical therapies [26].

There are no studies using carbolic acid to treat BPF with the help of BFS at present. Therefore, we investigated the efficiency of carbolic acid treatment of BPF in post-pulmonectomy patients, by instilled 100 % carbolic acid with the aid of BFS.

## Methods

### Ethics statement

This study was conducted with the approval of the Institutional Review Board of Liaoning Tumor Hospital, Shenyang. The informed written consent was collected from each eligible patient and the whole study was performed based on the Declaration of Helsinki [14].

### Study population

A total of 12 patients with post-pulmonectomy BPF were enrolled at the Department of Thoracic Surgery, Liaoning Tumor Hospital, Shenyang between February 2009 and March 2012. Orificium fistulae were confirmed by bronchoscope and the average diameter was 4.5 mm. The eligible patients included eight males and three females, with an average age of 56 years (range, 45 ~ 71 years). Three patients had BPF after the right pneumonectomy, six after the left pneumonectomy, one after the right middle and low lobectomy and two after left upper lobectomy.

### Preoperotive preparation

Electrocardiogram, routine blood tests and biochemical examination were performed in all the patients. Patients were fasted for 4 ~ 6 h in preparation for surgery and received 10 mg diazepam and 1 mg atropine via intramuscular injection about 30 min before operation. In addition, 1 % lidocaine was used for nasopharyngeal anesthesia by nebulizer.

### Intraoperative methods

All patients were instructed to take supine position except 2 patients with short breath in sitting position. The BFS (Olympus BF1T40) was inserted into the trachea through nasal cavity. Heart rate, blood pressure and SpO2 was monitored. Patients received local nasopharyngeal anesthesia with 2 % lidocaine to alleviate irritant reaction. The bronchus around the suture was bubbling when the patient breathed deeply. The fistula was observed via BFS. After the drainage of secretion, hematocele or pus around the BPF, a bronchoscopy biopsy forceps was used for removing necrotic tissues and a 1.8 mm flexible tube was guided through the biopsy hole. The distal end of BFS was brought out and fixed 0.3 cm above the fistula. With breath holding, 100 % carbolic acid solution (0.5–1.0 ml) was instilled to bronchial mucosa through the BFS. The bronchial mucosa became pale after treatment and finally the flexible tube and bronchoscope were removed.

### Postoperative and histological observation

After the surgery, the patients were treated with closed drainage of thoracic cavity, anti-inflammatory, symptomatic and supportive treatments. Gas discharge in thoracic drainage tube was observed, and fistula healing were measured via BFS. The treatments were repeated if there was gas discharge from thoracic drainage tube, or further observations were made. Patients could leave hospital after blood routine test showing no evidence of dyspnea, fever, positive culture of fluid drainage (3 times). Paraffin sections (4 ~ 6 μm) of bronchial stump were stained by hematoxylin and eosin (HE) to observe the irritation of bronchial stump after instilled with carbolic acid solution.

## Results

### Outcome characteristics of BPF

**Fig. 1:**
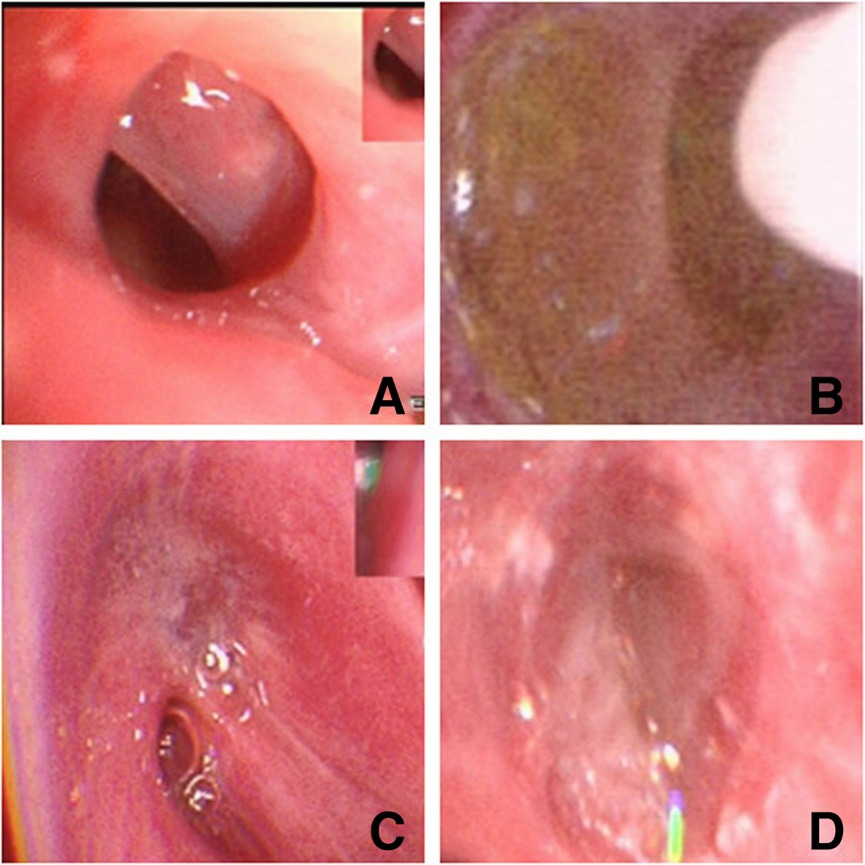
The treatment process of carbolic acid: **a** the BPF (about 1 mm); **b** infused by the carbolic acid; **c** BPF narrowed after 2 times instillation, and bubbles appeared after carbolic acid infused with saline; **d** repositioned the fistula, and three times later the BPF healed. Note: BPF, bronchopleural fistula

In the 12 patients with BPF, the median diameter of the BPF orifice was 4.5 mm, according to the intraoperative observation. Specifically, 3 patients showed a fistula diameter of 3 mm or smaller, 6 patients showed a fistula diameter of 3 ~ 5 mm, and 3 patients exhibited a fistula diameter of 5 mm or larger, 1 of whom had a fistula diameter of 7 mm (Table 1). Serious complications, such as haemorrhage, severe dyspnea and SpO2 declines, did not occur in all the 12 patients during bronchoscopic therapy. Of note, BPF orifices in 5 patients closed after 5 treatments with carbolic acid, 1 patient through 2 treatments, 1 patient through 3 treatments, 2 patients through 4 treatments and 3 patients through 7 treatments (Fig. 1). Follow-up was conducted for six months after bronchoscopy. Based on the data collected, the average treatment time of the 12 patients was calculated as 20 min and the average time of fistula closure was 30 days. Importantly, the cure rate was 100 %.

**Table 1:** Characteristics and outcomes of BPF patients

	Age (y)	Gender	Initial symptom	Surgical method	Bronchopleural fistulae			
					Size (mm)	Treatment times	Cure time (d)	Follow up
1	48	male	Low fever	Right PNY	3	5	35	alive
2	56	male	High fever	Right PNY	3.5	5	35	alive
3	50	male	Blood sputum	Left PNY	1	3	21	alive
4	71	male	Irritating cough	Left upper LBY	1.5	4	28	alive
5	57	female	Low fever	Right upper LBY	4	5	35	alive
6	65	male	Fever/air bubble	Left PNY	5	7	49	unknown
7	64	male	Cough/fever	Left PNY	7	7	49	alive
8	59	male	Cough/fever	Right PNY	1	2	14	alive
9	62	male	Low fever	Left upper LBY	4	7	49	alive
10	58	male	Fever/sputum	Left PNY	5	5	35	alive
11	45	male	Low fever	Left PNY	3.5	4	28	alive
12	56	male	sputum	Left PNY	4	5	35	alive

*BPF* bronchopleural fistula, *y* years, *PNY* pneumonectomy, *LBY* lobectomy, *d* days

### HE staining

**Fig. 2:**
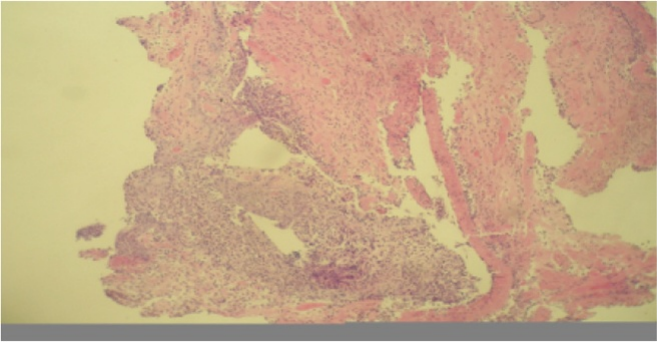
Results of HE staining observing bronchial stumps tissues stimulated by carbolic acid infusion. Note: HE, hematoxylin and eosin

HE staining was performed to observe bronchial stumps stimulated by carbolic acid infusion. The biopsy showed that the white flat hyperplasia tissue (bronchial stumps tissue) after carbolic acid treatment was inflammatory granulation tissue. Furthermore, it was found that the tissue was loose and dropsical, with a small amount of proliferation of irregularly distributed fibroblast, small vascular proliferation, a large number of plasma cells and lymphocyte infiltration, and a small amount of irregularly arranged squamous epithelial hyperplasia, as shown in Fig. 2.

## Discussion

BPF is defined as an abnormal communication between a lobar or the main bronchus and the pleural space, and continues to be a severe surgery complication, which is related to high morbidity and mortality [13]. Risk factors associated with BPF incidence are fever, steroid use, anemia, leukocytosis and tracheostomy, elevated erythrocyte sedimentation rate, Haemophilus influenzae in sputum and bronchoscopy for sputum suction or mucus plugging [2]. Recently, a number of flexible bronchoscopic techniques have been used to seal BPFs. These materials include cyanoacrylate-based glues, absorbable gelatine sponge, vascular embolisation devices, and fibrin compounds [7, 10]. In this study, we describe a novel approach of using carbolic acid for the closure of fistulas. Carbolic acid has a strong reaction with mucosal tissues, and pure carbolic acid corrodes mucosa completely in 60 s. When carbolic acid contacts the mucosal surface, the mucosa tissues is rapidly degenerated (pale), and mucosal inflammation stimulate exudation and proliferation, finally resulting in the closure of fistula [34]. Carbolic acid is widely used for disinfecting appendiceal stump in appendicitis operation and in the treatment of suspected TB contaminants in tuberculosis surgery [5, 17].

We describe a simple, safe and effective way to instill 100 % carbolic acid through BFS in the treatment of BPF. The 12 patients treated with carbolic acid successfully reached fistula closure, with the total effective rate at 100 % without any adverse reactions of hemoptysis and dyspnea. A reasonable explanation might be that carbolic acid is relatively safe, a small amount of acid liquid overflow will not cause serious injuries to normal mucosal [29]. A number of advantages are embodied in the instillation of carbolic acid under BFS. First, this method is easy to perform and, based on it success rate in this study, likely to be readily accepted by the patients, and patient hospitalization is unnecessary if they are in good condition. Second, for patients who have larger fistula with significant pleural effusion and sputum, this therapeutic tool can rapidly relieve the symptoms and avoid aspiration pneumonia. Third, the fistula location, size, shape can be clearly orientated. Finally, it can help reduce operative risks, decrease mortality rate as well as the cost of treatment for BPF [13]. Our results also revealed that the healing time of fistula is positively correlated to its size. Based on the clinical observations of series of cases, we summarize that fistula < 5 mm healed in much shorter time compared to fistula ≥ 5 mm. However, if the size of fistula exceeded certain limit, it might be difficult to heal due to the potential lack of healthy mucosa to stimulate proliferation and regenerate tissue [23].

Our study presents clear evidence that use of carbolic acid for BPF treatment under the inspection of BFS is safe and 100 % effective. However, our findings need to be interpreted with caution due to limitations in the study. A limitation is the small number of patients with BPF who underwent BFS. Therefore, our study contained a relatively smaller sample size, which might restrict the application of our results to a wider population. Further studies using large sample size and better study designs will be necessary to confirm our findings.

## Conclusion

In conclusion, we achieved 100 % efficacy in treatment of BPF with carbonic acid through BFS, with BPF size ranging from 3–7 mm in diameter. The described procedure is simple, safe and an effective choice for BPF patients, with little pain and at relatively low cost.
